# A critical assessment of matching-adjusted indirect comparisons in relation to target populations

**DOI:** 10.1017/rsm.2025.10

**Published:** 2025-03-21

**Authors:** Ziren Jiang, Jialing Liu, Demissie Alemayehu, Joseph C. Cappelleri, Devin Abrahami, Yong Chen, Haitao Chu

**Affiliations:** 1 School of Public Health, Division of Biostatistics and Health Data Science, University of Minnesota, Minneapolis, MN, USA; 2 Statistical Research and Data Science Center, Pfizer Inc., New York, NY, USA; 3 Global Access and Value, Pfizer Inc., New York, NY, USA; 4 Department of Biostatistics, Epidemiology and Informatics, University of Pennsylvania, Philadelphia, PA, USA; 5 The Center for Health AI and Synthesis of Evidence (CHASE), University of Pennsylvania, Philadelphia, PA, USA

**Keywords:** health technology assessment, indirect treatment comparison, MAIC paradox, matching-adjusted indirect comparison, population-adjusted indirect comparison

## Abstract

Matching-adjusted indirect comparison (MAIC) has been increasingly applied in health technology assessments (HTA). By reweighting subjects from a trial with individual participant data (IPD) to match the summary statistics of covariates in another trial with aggregate data (AgD), MAIC enables a comparison of the interventions for the AgD trial population. However, when there are imbalances in effect modifiers with different magnitudes of modification across treatments, contradictory conclusions may arise if MAIC is performed with the IPD and AgD swapped between trials. This can lead to the “MAIC paradox,” where different entities reach opposing conclusions about which treatment is more effective, despite analyzing the same data. In this paper, we use synthetic data to illustrate this paradox and emphasize the importance of clearly defining the target population in HTA submissions. Additionally, we recommend making de-identified IPD available to HTA agencies, enabling further indirect comparisons that better reflect the overall population represented by both IPD and AgD trials, as well as other relevant target populations for policy decisions. This would help ensure more accurate and consistent assessments of comparative effectiveness.

## Highlights

**What is already known:**

- Matching-adjusted indirect comparison (MAIC) methods are increasingly used in health technology assessment (HTA) submissions to adjust for population differences.
- MAIC estimates the comparative effectiveness of interventions for the population represented by the trial with aggregate data (AgD).

**What is new:**

- We present an illustration demonstrating an MAIC paradox in which the comparative effectiveness conclusions are reversed by switching the availability of IPD and AgD while adjusting the same set of effect modifiers.
- Additionally, we examine how variations in covariate distribution overlap of effect modifiers between trials influence the estimated comparative effectiveness.

**Potential impact for Research Synthesis Methods readers:**

- Through an illustrative example, we emphasize the vital importance of clearly defining the target population when applying MAIC in HTA submissions.
- We recommend providing de-identified IPD to HTA agencies to enable a more accurate assessment of comparative effectiveness for the target population.

## 1 Background of MAIC

Effect modification occurs when the magnitude of the effect of a treatment on an outcome differs depending on the value of a third variable. For example, studies indicate that Black individuals may experience less favorable outcomes compared to non-Black individuals when treated with angiotensin-converting enzyme (ACE) inhibitor-based therapies.^1^ ^,^ ^2^ In health technology assessments (HTAs), pharmaceutical companies are required to benchmark their new drugs against the prevailing standard of care for reimbursement decisions by HTA agencies.^3^ Nevertheless, the presence of effect modifiers can pose a unique challenge in comparing treatments when there is a lack of head-to-head trials. The traditional Bucher method,^4^ which compares the relative treatment effects of two interventions assessed in two randomized trials without covariate balancing, is limited to scenarios where all effect modifiers are balanced across trial populations.^5^ When individual participant data (IPD) are available for one trial while aggregate-level data (AgD) are only available for the other trial, researchers introduced population-adjusted indirect comparison (PAIC) methods to obtain unbiased estimates of comparative effectiveness, particularly when imbalances in effect modifiers exist. PAIC methods include matching-adjusted indirect comparison (MAIC),^6^ simulated treatment comparison (STC),^7^ and multilevel network meta-regression (ML-NMR).^8^

Among these methods, MAIC is becoming increasingly popular and widely used in health technology appraisals, such as the National Institute for Health and Care Excellence (NICE) in the United Kingdom.^9^ In a recent methodological systematic review, 88.9% (144 out of 162) of PAIC studies used MAICs.^10^ MAICs estimate a set of balancing weights for each subject in the IPD trial such that the weighted summary statistics (e.g., mean and standard deviation) of covariates in the IPD trial match the reported summaries of the same covariates in the AgD trial. Then, MAIC compares the marginal treatment effect estimated using the weighted data in the IPD trial with the marginal treatment effect reported in the AgD trial.^5^ ^,^ ^11^ For a more detailed description of the MAIC methods, readers can refer to the review paper by Jiang et al.^12^

Notably, the indirect comparison result is only valid with respect to the population represented by the AgD trial. However, this may not align with the population of interest for the company conducting the MAIC, which might prioritize the population represented by the IPD trial or another specific target population. This paper presents an illustrative example showing that MAICs can yield conflicting comparative effectiveness results when switching the availability of AgD and IPD between trials. This paradoxical phenomenon occurs due to differing magnitudes of effect modification for the two drugs by an effect modifier that is also imbalanced between the trial populations.

## 2 An illustrative example of the MAIC paradox

**Figure 1:**
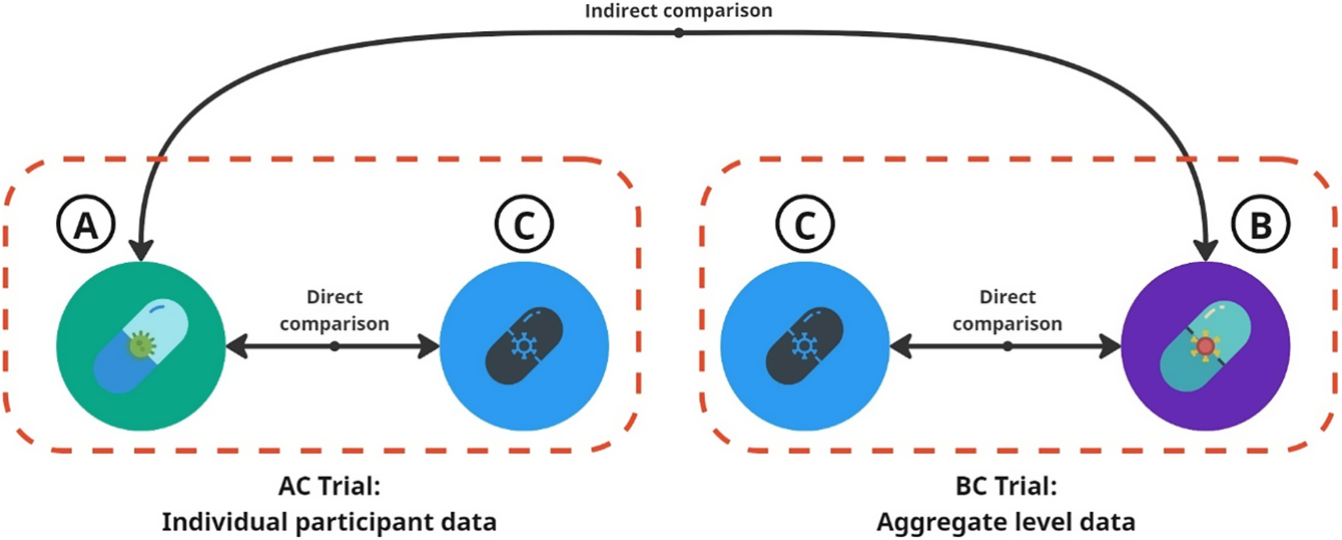
*Indirect comparison of Drug A versus B in two trials. For the AC trial, we have the individual participant data (IPD). For the BC trial, we only have the aggregate level data (AgD)*.

Consider an anchored indirect comparison between drug A (from Company A) and drug B (from Company B), each compared to a common placebo comparator C, as depicted in Figure 1. Each company has access only to IPD from its own trial and to AgD for the other company’s trial through published sources. For simplicity, we assume that race (Black versus non-Black) is the sole effect modifier, allowing both MAICs to include the “correct” effect-modifying variable. Additionally, we assume that drug A shows a stronger treatment effect among Black participants, while drug B is more effective among non-Black participants. If the AC trial includes a higher proportion of non-Black participants (among whom drug A is less effective than drug B), while the BC trial predominantly includes Black participants, we can observe a paradox: in separate MAICs, drug A outperforms drug B in the BC trial population, while drug B outperforms drug A in the AC trial population.

Table 1 presents hypothetical data of the AC and BC trials. Let 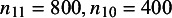 be the number of non-Black and Black patients in the AC trial, and 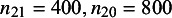 denote the number of non-Black and Black patients in the BC trial, respectively. The MAIC performed by Company A calculates the weights 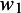 for all non-Black patients and 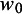 for all Black patients in the AC trial such that the weighted proportion of non-Black patients 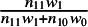 matches the proportion of non-Black patients in the BC trial 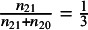 (see Table 1), subject to the constraint that weights sum to 1 (i.e., 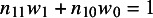 ). Solving the equation, we have 
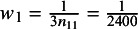
 and 
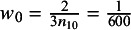
 . Based on the trial data, under the usual logit link, the estimated treatment effect (log of the odds ratio of the survival rate) for drug A versus drug C in the population of BC trial would be

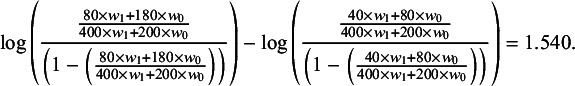

**Table 1:** Results for the illustrative example. In this example, the risk difference in survival rate for Drug A versus Drug C in the AC trial is 10% for non-black patients and 50% for black patients. Treatment effect for Drug B versus Drug C in the BC trial is 40% for non-black patients and 20% for black patients

		AC Trial			BC Trial			A versus B
		Drug A	Drug C	logOR^*^	Drug B	Drug C	logOR	logOR
Non-black	Y = 0^**^	80	40		100	20		
	Y = 1	320	360		100	180		
	n^***^	400	400		200	200		
	Survival rate	20%	10%	0.81	50%	10%	2.20	−1.39
Black	Y = 0	180	80		240	160		
	Y = 1	20	120		160	240		
	n	200	200		400	400		
	Survival rate	90%	40%	2.60	60%	40%	0.81	1.79
Overall	Survival rate	43.3%	20%	1.12	56.7%	30%	1.12	

* logOR: Log of odds Ratio.

** Y: Outcome variable with Y = 1 indicating death and Y = 0 indicating survival.

*** n: Sample size.

The indirect comparison of drug A versus B can then be obtained by subtracting the estimated marginal treatment effect for drug B in the BC trial from this value, which gives 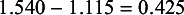 . The corresponding standard error can be calculated using the robust sandwich estimator, which provides robust results given that the weights are estimated. The 95% confidence interval can then be constructed as 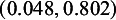 , indicating that drug A is statistically significantly better than drug B in the population of the BC trial.

Similarly, for the MAIC performed by Company B with IPD and AgD switched, the weights can also be determined by matching the weighted proportion of non-Black patients in the BC trial (IPD trial) to the proportion in the AC trial (AgD trial). This gives 
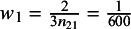
 and 
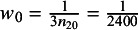
 . Performing the same calculations, the estimated comparative effectiveness for drug A versus drug B in the population of the AC trial is 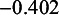 with a 95% confidence interval 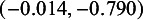 , which indicates that drug B is significantly better than drug A in the population of the AC trial.

Here, we emphasize that both conclusions—drug A being more effective than drug B or B being more effective than A—are potentially valid within the context of the specific populations considered in this example. However, without a clearly defined target population, the results of the indirect comparison lack meaningful applicability for guiding medical decisions.

In the supplementary material Section S1, we show a step-by-step derivation of the weights. In Section S2, we further explore the impact of varying proportions of non-Black participants in the IPD and AgD populations separately on the MAIC results (see Figures S1a and Figure S1b). In addition, we present an example illustrating this paradox in unanchored MAIC in Section S3.

## 3 Discussion

In this manuscript, we highlight a potential paradox, referred to as the “MAIC paradox,” where both companies may claim the superiority of their drugs through MAIC, even when the same covariates, including all effect modifiers, are included in the analyses. This paradox arises when there are imbalances in effect modifiers with different magnitudes of modification across treatments. The key issue is the lack of careful consideration of the target population. If the target population is not clearly defined or appropriately selected for the context, results from MAIC may lead to misleading or contradictory conclusions. This emphasizes the need for Health Technology Assessment (HTA) appraisals to explicitly define the target population that is most relevant for policy decision-making to ensure valid and consistent results. A clear definition of the target population is crucial to avoid misinterpretations that may arise from the MAIC paradox. Additionally, population overlap can impact MAIC results. Indirect comparison results tend to be more consistent when there is a high degree of overlap between populations. Conversely, if neither the AC trial population nor the BC trial population is comparable to the target population, MAIC may not reliably estimate the most relevant treatment effect. A more detailed discussion is provided in Supplementary Material Section S2 .

The dependence of MAIC results on the target population has also been discussed in other literature as well. Following a review of NICE appraisals, Phillippo et al.^9^ observed that many appraisals overlooked the fact that comparative effectiveness was estimated over the population from the AgD trial, which might not represent the target population of interest. An example provided by the NICE DSU Technical Support Document 18^11^ illustrates how contradicting conclusions may arise when both Novartis and AbbVie used MAIC to compare their drugs secukinumab and adalimumab: Novartis claimed significant efficacy advantages for secukinumab, while AbbVie argued that adalimumab had comparable efficacy but was more cost-effective. This discrepancy arose in part because two MAICs used different sets of covariates in their analyses, which complicated the understanding of the cause for inconsistency. In our manuscript, we show that such paradoxes can still occur even when the correct MAIC model is used with the same set of covariates.

The manuscript illustrates how the target population affects the results of MAIC. However, the results of other PAIC methods, such as STC, also depend on the target population. Additionally, extrapolating outside the IPD population increases the risk of bias in the case of STC, which is a broader concern for any regression-based or other adjustment method. In the presence of effect modifiers, indirect comparison results are only valid when the target population is clearly defined.

Phillippo et al.^5^ proposed an additional sufficient condition for validly extrapolating the comparative effectiveness results to other populations, known as the “shared effect modifier assumption.” This assumption has two key components: 1) treatment effect modifiers are the same for all treatments, and 2) the magnitude of each effect modifier (i.e., how much it influences the treatment effect) is the same for all included treatments. Under the classical two-trial scenario, this assumption is statistically untestable, as only one trial provides IPD, leaving the other trial’s effect modifiers uncertain. Therefore, the validity of this assumption must only be demonstrated from a clinical perspective, relying on existing knowledge about the disease and treatments. This highlights the importance of ensuring clinical consistency and understanding of the treatments involved when applying MAIC or other PAIC methods.

Finally, we advocate for a collaborative effort among all relevant stakeholders to make de-identified IPD from clinical trials available through a trusted authority. Access to IPD from both trials would allow for a more robust approach to balancing the entire covariate distribution rather than just balancing the moments (such as means) of the covariates. This help mitigate the risk of ecological fallacy when drawing inferences between the two populations. However, sharing data must be done with the informed consent of trial participants, ensuring that appropriate de-identification protocols are followed to minimize the risk of participant re-identification.^13^ In addition to direct sharing of IPD, interim solutions could include the use of federated learning algorithms^14^ ^–^ ^17^ and secure data-sharing infrastructures,^18^ which allows for the analysis of summary statistics without exposing sensitive individual data.

## Supporting information

Jiang et al. supplementary material Jiang et al. supplementary material

## Data Availability

The simulated data used in the illustrative example are presented in Table 1.

## Supplementary material

To view supplementary material for this article, please visit http://doi.org/10.1017/rsm.2025.10.
