# Supplementary material for: A critical assessment of matching-adjusted indirect comparisons in relation to target populations
Source: Res Synth Methods. 2025 Mar 21;16(3):569–74. doi: 10.1017/rsm.2025.10 (PMC12527493; doi:10.1017/rsm.2025.10)
Supplement: Jiang et al. supplementary material [file S1759287925000109sup001.pdf]

Supplementary Material

S1. Step-by-step derivation of the MAIC weights

Here we give a step-by-step derivation on the MAIC weights for incorporating a single categorical variable X with two categories. Without loss of generality, assume $X \in \{0, 1\}$. Assume the proportion of $X = 1$ in the population of aggregate data trial is $\mu \in [0,1]$. For the trial with individual participant data, assume the number of participants with $X = 1$ is n_1 and the number of participants with $X = 0$ is n_0. Then, the MAIC estimates two balancing weights (since the variable has only two categories), w_1 and w_0 such that: 1) The weighted proportion of $X = 1$ in the IPD trial population equals to μ, i.e.,

$$\frac{n_1 w_1}{n_1 w_1 + n_0 w_0} = \mu$$

and 2) the weights sum to 1, i.e.,

$$n_1 w_1 + n_0 w_0 = 1.$$

Solving the two equations, we have:

$$w_1 = \frac{\mu}{n_1}, \text{and}$$

$$w_0 = \frac{1-\mu}{n_0}.$$

Note that, this requires that both n_1 and n_0 are larger than 0. Otherwise, the IPD trial population only has one type of participants and there is no way to balance the distribution.

We further note that, since the covariate under adjustment is a categorical variable with 2 categories, the estimated weights only have two degrees of freedom with two equations solving them. This makes the weights unique for the MAIC with method of moment and MAIC with largest effective sample size, if a solution exists (i.e., $n_1, n_0 \neq 0$). This assertion is also applicable for the categorical variable with K categories where we would have K degrees of freedom for the weights (i.e., $w_1, \dots, w_K$) and K equations corresponding to it. If a solution exists for the weights (i.e., there is at least one observation for each category of the IPD trial), it is unique.

S2: Exploration of the impact of varying proportions of non-Black participants

We further vary the proportion of non-Black participants in the IPD and AgD populations separately to explore the impact on MAIC results, using the same settings as the illustrative example above. In Figure S1a, we vary the proportion of non-Black participants in the AgD trial while keeping it fixed it in the IPD trial. Because the MAIC specifically targets the AgD trial population, changing the proportion of non-Black participants in the AgD trial led to substantial changes in the estimated treatment effects. In Figure S1b, we vary the proportion of non-Black participants in the IPD trial population while maintaining a constant non-Black proportion in the AgD trial. Since the same population is targeted regardless of the covariate distribution in the IPD trial, MAIC produces the same results despite fluctuations in the IPD trial population. We also present the effective sample size (ESS) for the MAIC weights under different proportions of the non-Black participants. As shown in the figure, the ESS is largest when the IPD trial population closely matches the AgD trial population. However, significant differences between the two populations result in a smaller ESS, which increases the uncertainty of the estimates.

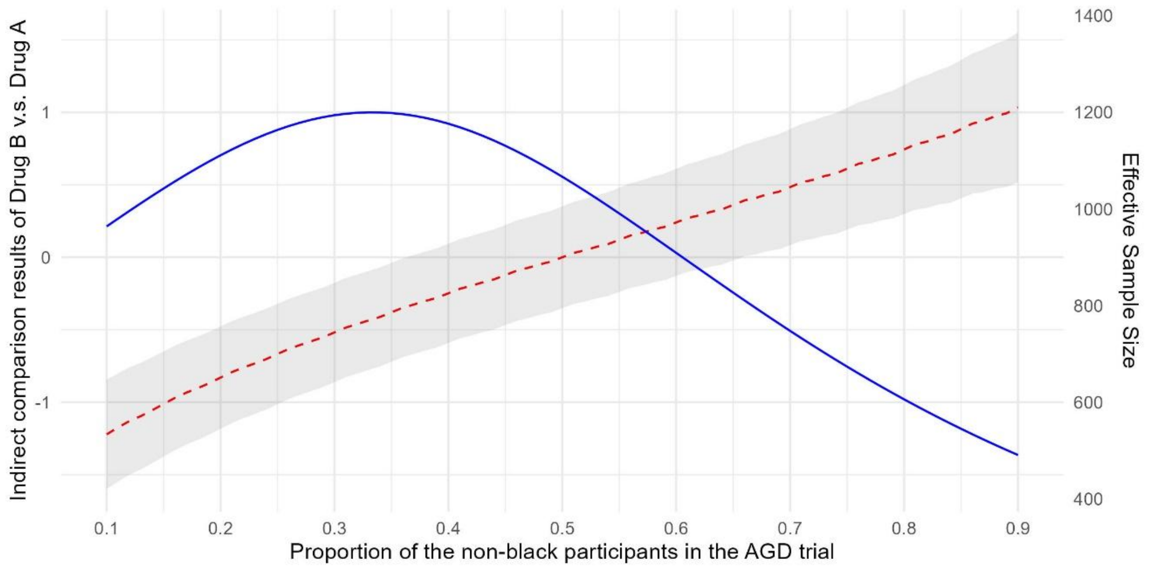

Figure S1a: MAIC results with varying proportions of non-Black participants in the AgD trial, while the IPD trial fixed at a proportion of 1/3 non-Black participants. We assume the same setting as in the illustrative example where the race of participants is the only effect modifier. Drug B is assumed to perform better than Drug A among non-Black participants, while Drug A performs better among black participants. The red dashed curve represents the estimated comparative effectiveness on the log-odds ratio scale, and the shaded region indicates the corresponding 95% confidence interval. The blue solid curve represents the effective sample size, defined as $\frac{(\sum_i w_i)^2}{\sum_i {w_i}^2}$, while w_i are the estimated weights.

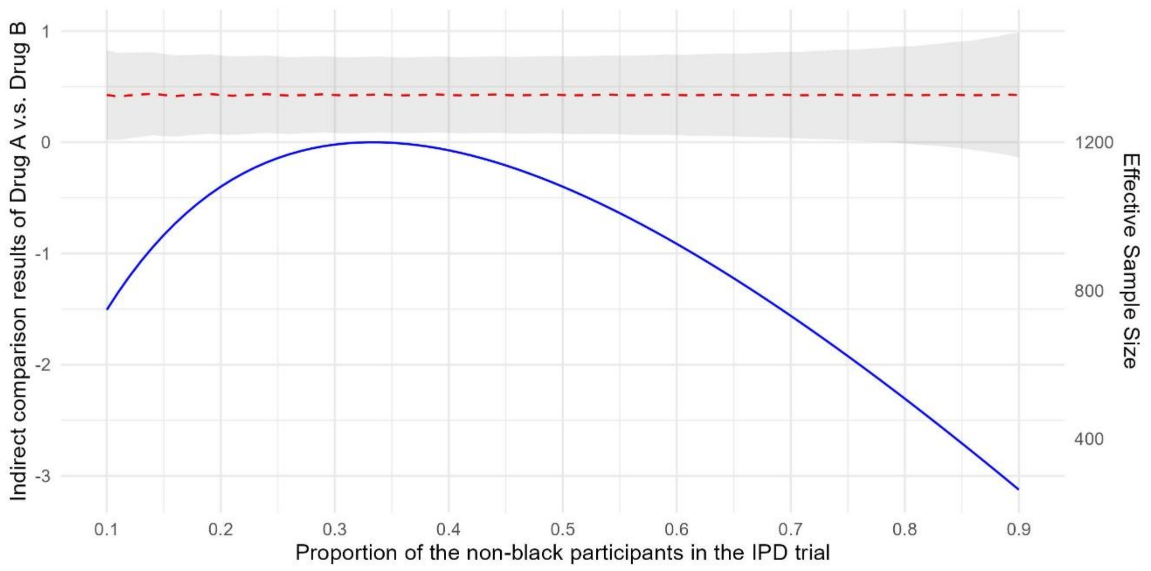

Figure S1b: MAIC results with varying proportions of non-Black participants in the IPD trial while the AgD trial fixed at a proportion of 1/3 non-Black participants. We assume the same setting as in the illustrative example where race of participants is the sole effect modifier. Drug B is assumed to perform better than Drug A among non-black participants, while Drug A performs better among black participants. The red dashed line represents the estimated comparative effectiveness on the log-odds ratio scale, and the shaded region depicts the corresponding 95% confidence interval. The blue solid curve represents the effective sample size, defined as $\frac{(\sum_i w_i)^2}{\sum_i {w_i}^2}$, where w_i are the estimated weights.

S3. Example of the paradox for an unanchored MAIC

In this section, we introduce another example of the paradox in the unanchored MAIC. See Table S1 for the results of drug A and drug B among Black and non-Black patients. Like the example in the manuscript, we assume that the binary variable of race is the only prognostic variable, so the unanchored MAIC fits the "correct" model by including the variable of race. For Black patients, drug A has a survival rate of 90% and drug B has a survival rate of 60%. For non-Black patients, drug A has a survival rate of 20% while drug B has a survival rate of 50%. Thus, drug A has a better effect than drug B among Black patients, while drug B is more effective than drug A among non-Black patients. If we have the IPD for the trial of drug A and only the AgD for the trial of drug B, the estimated weights will be $w_1 = \frac{1}{3\times 400} = \frac{1}{1200}$ for non-Black patients and $w_0 = \frac{2}{3\times 200} = \frac{1}{300}$ for Black patients. The estimated comparative effectiveness (log-odds ratio) of drug A versus drug B is 0.424 with a standard error 0.124. Similarly, if we have the IPD for the trial of drug B and only the AgD for the trial of drug A, the estimated comparative effectiveness of drug B versus drug A will be 0.401 with a standard error 0.130.

Table S1: Results for the illustrative example of unanchored MAIC. In this example, the survival rate under Drug A is 20% for non-Black patients and 90% for Black patients. The survival rate under Drug B is 50% for non-Black patients and 60% for Black patients in the population of Trial B. Like the illustrative example in the manuscript, we also provided the log of odds ratio corresponding to the survival rates.

		**Drug A**	**Drug B**	**A vs. B RD (logOR)**
**Black**	**Y=0**	180	240	
	**Y=1**	20	160	
	**n**	200	400	
	**Survival rate**	90%	60%	30% (1.79)
**non-Black**	**Y=0**	80	100	
	**Y=1**	320	100	
	**n**	400	200	
	**Survival rate**	20%	50%	-30% (-1.38)
